# Genetic variation of *circHIBADH* enhances prostate cancer risk through regulating HNRNPA1-related RNA splicing

**DOI:** 10.7555/JBR.38.20240030

**Published:** 2024-05-29

**Authors:** Yifei Cheng, Rongjie Shi, Shuai Ben, Silu Chen, Shuwei Li, Junyi Xin, Meilin Wang, Gong Cheng

**Affiliations:** 1 Department of Environmental Genomics, Jiangsu Key Laboratory of Cancer Biomarkers, Prevention and Treatment, Collaborative Innovation Center for Cancer Personalized Medicine, Nanjing Medical University, Nanjing, Jiangsu 211166, China; 2 Department of Urology, the First Affiliated Hospital of Nanjing Medical University, Nanjing, Jiangsu 210029, China; 3 Department of Bioinformatics, School of Biomedical Engineering and Informatics, Nanjing Medical University, Nanjing, Jiangsu 211166, China; 4 Jiangsu Cancer Hospital, Jiangsu Institute of Cancer Research, the Affiliated Cancer Hospital of Nanjing Medical University, Nanjing, Jiangsu 210009, China; 5 The Affiliated Suzhou Hospital of Nanjing Medical University, Suzhou Municipal Hospital, Gusu School, Nanjing Medical University, Suzhou, Jiangsu 215002, China

**Keywords:** genetic variants, prostate cancer, circRNA, RNA-binding protein, RNA splicing, sing-cell RNA sequencing

## Abstract

The current study aimed to investigate associations of circRNAs and related genetic variants with the risk of prostate cancer (PCa) as well as to elucidate biological mechanisms underlying the associations. We first compared expression levels of circRNAs between 25 paired PCa and adjacent normal tissues to identify risk-associated circRNAs by using the MiOncoCirc database. We then used logistic regression models to evaluate associations between genetic variants in candidate circRNAs and PCa risk among 4662 prostate cancer patients and 3114 healthy controls, and identified *circHIBADH* rs11973492 T>C as a significant risk-associated variant (odds ratio = 1.20, 95% confidence interval: 1.08–1.34, *P* = 7.06 × 10 ^−4^) in a dominant genetic model, which altered the secondary structure of the corresponding RNA chain. In the *in* *silico* analysis, we found that *circHIBADH* sponged and silenced 21 RNA-binding proteins (RBPs) enriched in the RNA splicing pathway, among which HNRNPA1 was identified and validated as a hub RBP using an external RNA-sequencing data as well as the in-house (four tissue samples) and publicly available single-cell transcriptomes. Additionally, we demonstrated that HNRNPA1 influenced hallmarks including MYC target, DNA repair, and E2F target signaling pathways, thereby promoting carcinogenesis. In conclusion, genetic variants in *circHIBADH* may act as sponges and inhibitors of RNA splicing-associated RBPs including HNRNPA1, playing an oncogenic role in PCa.

## Introduction

Prostate cancer (PCa) is the second most common malignancy and the fifth leading cause of cancer-related deaths in men ^[ 1] ^, with a good prognosis and a 5-year survival rate of over 98% ^[ 2] ^. However, patients with advanced PCa have a much poorer prognosis due to the lack of effective therapies ^[ 3] ^. It was reported that a high heritability (approximately 57%) and an increasing risk were observed in men with a family history of PCa, implying a genetic component in the etiology of this disease ^[ 4] ^. Genome-wide association studies have identified more than 200 loci associated with PCa risk ^[ 5] ^, but approximately 90% of these susceptibility loci are in non-coding regions. Moreover, the mechanisms underlying these associations remain unclear ^[ 6] ^.

Gene expression is regulated by numerous non-coding RNAs, including long non-coding RNAs, microRNAs, and circular RNAs (circRNAs), with a particular attention paid to the role of circRNAs in cancers. It is known that circRNAs are mainly formed by back-splicing events that splice an exon into an upstream one ^[ 7] ^. The circRNAs may function in biological processes *via* sponging microRNAs ^[ 8] ^, interacting with RNA-binding proteins (RBPs) as sponges ^[ 9] ^, scaffolds ^[ 10] ^, recruiters ^[ 11] ^, protein function enhancers ^[ 12] ^, and translating proteins ^[ 13] ^. Through these functions, circRNAs play either oncogenic or tumor suppressor roles in various malignancies, including cancers of the bladder ^[ 14] ^, stomach ^[ 15] ^, breast ^[ 16] ^, and prostate ^[ 17– 18] ^. Using an exome capture RNA sequencing protocol, Vo *et* *al* ^[ 19]^ detected 160120circRNAs in more than 2000 cancer samples and found a general downregulation of these circRNAs in tumor tissues, which is attributed to the regulation of cellular proliferation. Additionally, they established the MiOncoCirc database to provide a resource of circRNAs for developing diagnostic targets in cancer. Moreover, Chen *et* *al* ^[ 17]^ discovered in several cohorts that circRNA expression levels were correlated with PCa progression. However, few studies have focused on the effects of genetic variants of circRNAs on the risk of PCa.

Therefore, the current study comprehensively investigated associations of genetic variants in circRNAs with PCa risk and survival. Furthermore, we evaluated how these genetic variants affect circRNAs and their functioning in binding with RBPs and regulating subsequent biological processes.

## Subjects and methods

### Study population and data processing

The current study included 4662 PCa patients and 3114 controls from the Prostate, Lung, Colorectal, and Ovarian (PLCO) Cancer Screening Trial with approval from the PLCO consortium (PLCO-84), which are available from NIH-CDAS ( https://cdas.cancer.gov/datasets/plco/20/). The baseline characteristics of the study population from the PLCO trial have been described previously ^[ 20] ^. In addition, we used the MiOncoCirc database that includes 25 pairs of matched PCa tumor and adjacent normal tissues ^[ 19]^ by downloading the expression matrices of circRNAs and their parental genes from the website ( https://mioncocirc.github.io/). Furthermore, we used RNA expression profiles and clinical information from The Cancer Genome Atlas (TCGA) prostate adenocarcinoma (PRAD) as well as GSE94767 and GSE183019 datasets from the Genomic Data Commons ( https://portal.gdc.cancer.gov/) and Gene Expression Omnibus ( https://www.ncbi.nlm.nih.gov/gds), respectively. The mRNA expression data from TCGA were transformed to values in transcripts per million before analysis. In the validation, we used additional single-cell RNA-sequencing (scRNA-seq) data of four PCa tissues from the First Affiliated Hospital of Nanjing Medical University, along with previously published single-cell transcriptome dataset ( https://www.prostatecellatlas.org/) ^[ 21] ^.

### Selection of circRNAs and single nucleotide polymorphisms (SNPs)

By comparing the expression levels of 25 paired tumor and adjacent normal tissues, we selected the risk-associated circRNAs based on the following inclusion criteria: (1) detected in circBase; (2) located on autosomal chromosomes; (3) a false discovery rate (FDR) < 0.01; (4) |log _2_(fold change [FC])| > 1; and (5) a call rate in normal tissues > 60%.

For SNP selection, we first extracted SNPs within the circRNA regions from the PLCO trial for further filtering. The quality control was carried out by using the following inclusion criteria: a minor allele frequency ≥ 0.05, *P*_Hardy-Weinberg equilibrium_ ≥ 0.05, and a call rate > 95%. Subsequently, the *in* *silico* analyses were performed for functional annotation. SNPs with a RegulomeDB (v2.1, http://regulome.stanford.edu/) ranking ≤ 5 or a 3DSNP score (v1.0, https://www.omic.tech/3dsnp/) ≥ 10 were considered functional SNPs. Tagging SNPs were obtained by conducting a pairwise linkage disequilibrium analysis. Finally, the risk-associated analyses were performed to identify SNPs that were significantly associated with PCa risk by using a dominant genetic model.

### Functional analysis of circRNAs

Based on the overview of circRNAs provided by the Cancer-Specific CircRNA Database (CSCD, http://gb.whu.edu.cn/CSCD/), we focused on the potential RBPs that bind to candidate circRNAs using both the circAtlas database ( http://circatlas.biols.ac.cn/) and CSCD. The potential functions of predicted RBPs were annotated through over-representative analyses using the Kyoto Encyclopedia of Genes and Genomes pathway and gene ontology biological processes datasets. Furthermore, we applied String ( https://string-db.org/) and Cystoscope software (version 3.7.1) to construct a protein-protein interaction (PPI) network of these RBPs.

### Identification and validation of hub RBPs and key biological processes

Based on the TCGA PRAD dataset, we performed differential expression (DE) analyses to identify differences in the predicted RBPs between normal and tumor tissues. By integrating PPI network and DE analyses, we determined RBPs that were located at the hub of the network, consistently expressed among samples, and significantly differentially expressed between tumor and normal tissues as the hub RBPs.

Through correlation analysis of the hub RBPs with candidate circRNAs in MiOncoCirc, we validated the regulatory potential and correlations of circRNA to RBPs. Furthermore, we performed the DE analysis in two external bulk and two single-cell transcriptomes to verify the expression patterns of the hub RBPs. We then assessed the differences in gene expression levels (log _2_FC) between the top and the bottom 1/4 TCGA samples according to the expression levels of the hub RBPs for the Gene-Set Enrichment Analysis (GSEA) based on CancerSEA database ( http://biocc.hrbmu.edu.cn/CancerSEA/) to discover hallmarks that might be affected by these RBPs in the tumorigenic process. Finally, we validated the activation of key biological processes by comparing the AUCell score (an algorithm that uses the area under the curve [AUC] to evaluate the enrichment of a particular gene set among the expressed genes of each cell) of the corresponding gene set of single cells between groups.

### scRNA-seq

The fresh tumor tissues from 10 patients were stored in the GEXSCOPE ^®^ Tissue Preservation Solution (Singleron, Nanjing, China) and transported on ice, which were then washed with Gibco Hanks Balanced Salt Solution (Cat. #14175095, Thermo Fisher Scientific-CN, Shanghai, China), minced and digested with GEXSCOPE ^®^ Tissue Dissociation Solution (Singleron), centrifuged and resuspended. GEXSCOPE ^®^ red blood cell lysis buffer (Singleron) was used to remove the red blood cells. Single-cell suspensions were converted to barcoded scRNA-seq libraries using the Chromium Single Cell Library, Gel Bead & Multiplex Kit (10x Genomics, Pleasanton, CA, USA), following the manufacturer's instructions. Finally, libraries were prepared using the 10x Genomics Library Kits (10x Genomics) and sequenced on Illumina Nova6000 (Illumina, San Diego, CA, USA) with a paired-end 150 bp reading strategy.

### Interpretation of scRNA-seq data

Raw reads were processed to generate gene expression profiles using Cell Ranger (v.3.0.2, 10x Genomics). Reads from the 10× library were mapped to GRCh38 with ensemble version 92 gene annotation. Cells with gene counts less than 500, unique molecular identifier counts less than 1000, or more than 15% expression levels of mitochondrial genes were filtered out, with genes expressed in less than 10 cells also excluded from the analysis.

The retained cells were then subjected to the " *Seurat*" program (v.4.3.0) in R for downstream analyses. After normalizing gene expression, we identified the top 2000 variable genes that were scaled and regressed out for the expression percentage of mitochondrial genes and used for principal component analysis. The batch effect was assessed using the R package " *Harmony*" (v.0.1.1). We uniformed the Manifold Approximation and Projection dimensionality reduction on the Harmony dimensions to obtain two-dimensional representations for data visualization, and then computed the shared nearest neighbor to identify clusters by using Harmony dimensions. Epitheliums were labeled by a high expression of *CDH1*/ *KRT8* and a low expression of *PTPRC*, and extracted for subsequent analyses. The annotation of epitheliums was carried out according to the epithelial subtypes identified by Henry *et* *al* based on the identified clusters ^[ 22] ^.

### Statistical analysis

We used R software (version 4.3.1) and PLINK (version 1.90) for statistical analyses and performed the Wilcoxon test for all differential expression analyses. The differences in baseline and clinical characteristics between PCa cases and the controls in the PLCO were assessed using the Student's *t-*test and *χ* ^*2*^ test for continuous variables and categorical variables, respectively. The goodness-of-fit test was applied to compute the Hardy-Weinberg equilibrium based on the allele frequencies of the controls. Univariate and multivariate logistic regression models were used to perform the risk-associated analyses. Differences in the expression levels of individual mRNAs and circRNAs were compared using the Wilcoxon signed-rank test and Wilcoxon rank-sum test for paired and unpaired samples, respectively. Over-representative analyses and GSEA were carried out with the R package " *clusterProfiler*" (v.4.2.2). Correlation analyses were conducted using Spearman's correlation method. AUCell scores were computed using the R package " *AUCell*" (v.1.16.0).

## Results

### circRNAs and SNPs selection

**Figure 1:**
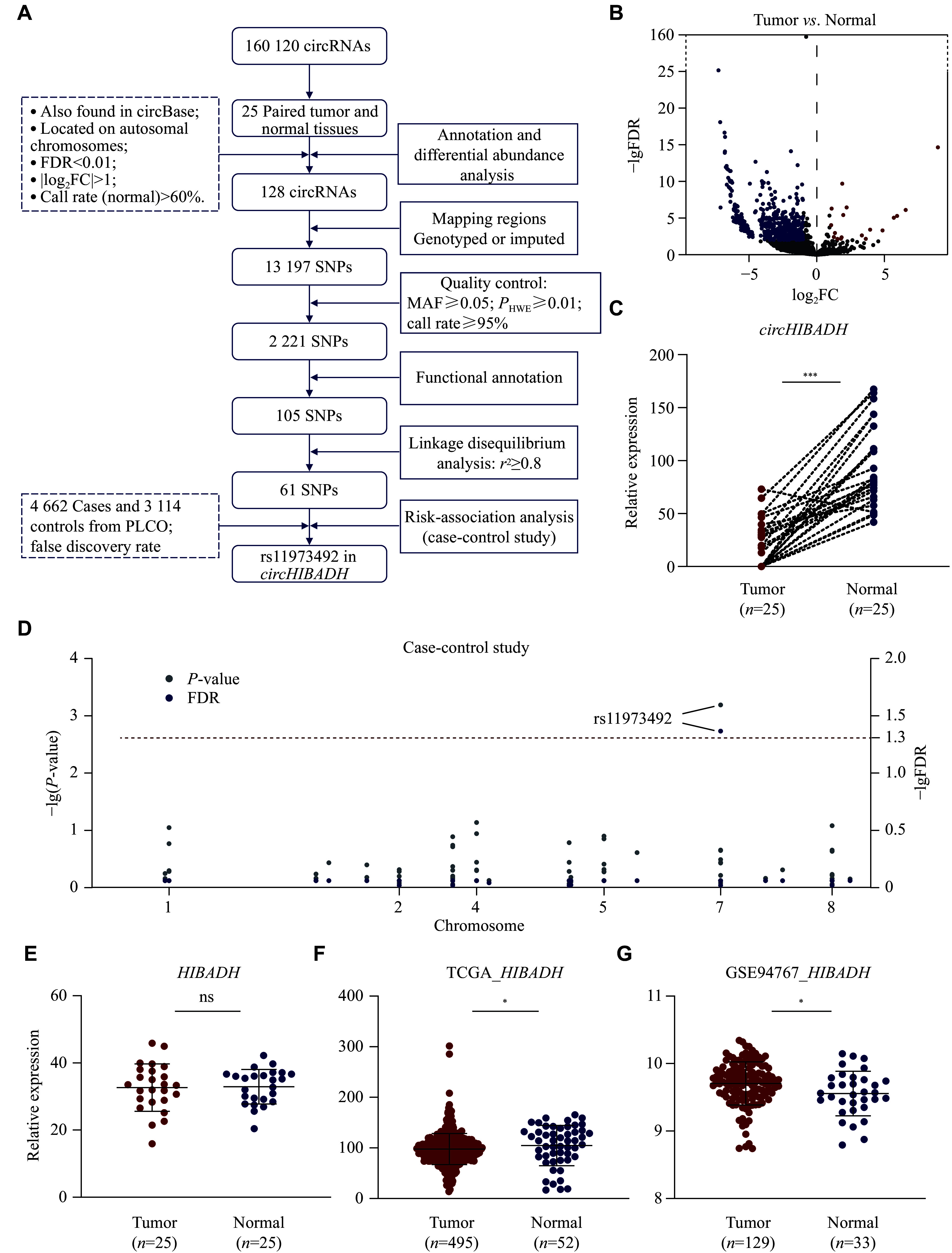
SNP selection among PCa-related circRNA regions.

The selection process of circRNAs and SNPs is shown in ***Fig. 1A***. Through differential expression analyses, 128 risk-associated circRNAs were identified ( ***Fig. 1B*** and ***Supplementary Table 1*** [available online]). In total, 13197 SNPs were included for quality control, functional annotation, and linkage disequilibrium analysis, resulting in the retention of 105 risk-associated SNPs for further analyses. SNPs were then analyzed for their associations with PCa risk. As shown in ***Supplementary Table 2*** (available online) and ***Fig. 1D***, only rs11973492 in *circHIBADH* (hsa_circ_0006773 in circBase), with an adjusted odds ratio (OR) = 1.20, 95% confidence interval (CI): 1.08–1.34, and FDR = 4.30 × 10 ^−2^, was significantly associated with PCa risk in a dominant genetic model.

### Associations between rs11973492 and PCa risk

The associations of rs11973492 with PCa risk in the co-dominant, additive, dominant, and recessive genetic models are shown in ***Table 1***. The proportions of TT, TC, and CC were 45.18%, 45.24%, and 9.58% in the cases, respectively, and 49.92%, 40.95%, and 9.13% in the controls, respectively. The TC genotype was significantly associated with an increased risk of PCa, compared with the TT genotype (adjusted OR = 1.23, 95% CI: 1.10–1.37, *P* = 3.07 × 10 ^−5^). A significantly higher risk of PCa was associated with TC/CC genotypes (adjusted OR = 1.20, 95% CI: 1.08–1.34, *P* = 7.06 × 10 ^−4^) in the dominant model but not in the recessive model (adjusted OR = 1.04, 95% CI: 0.91–1.18, *P* = 6.03 × 10 ^−1^).

**Table 1:** Associations between rs11973492 in *circHIBADH* and risk of prostate cancer

Genotypes	Cases ( *N*=4624)			Controls ( *N*=3079)		OR (95%CI)	*P*	Adjusted OR (95%CI) ^a^	*P* ^a^
	*n*	%		*n*	%				
TT	2089	45.18		1537	49.92	1		1	
TC (TC *vs*. TT)	2092	45.24		1261	40.95	1.22 (1.11–1.34)	4.72×10 ^−5^	1.23 (1.10–1.37)	3.07×10 ^−5^
CC (CC *vs*. TT)	443	9.58		281	9.13	1.16 (0.99–1.37)	7.50×10 ^−2^	1.09 (0.90–1.31)	3.95×10 ^−1^
Additive model (CC *vs*. TC *vs*. TT)						1.13 (1.05–1.21)	5.97×10 ^−4^	1.11 (1.02–1.21)	1.16×10 ^−2^
Dominant model (CC & TC *vs*. TT)						1.21 (1.10–1.33)	4.46×10 ^−5^	1.20 (1.08–1.34)	7.06×10 ^−4^
Recessive model (CC *vs*. TC & TT)						1.06 (0.90–1.23)	5.04×10 ^−1^	1.04 (0.91–1.18)	6.03×10 ^−1^
^a^Adjusted for age, smoking status, and the top ten principal components in the logistic regression model. Abbreviations: OR, odds ratio; CI, confidence interval.									

We further conducted stratification analyses for the association between rs11973492 and PCa by using the dominant model for clinical characteristics. As shown in ***Table 2***, TC/CC genotypes were risk factors in PCa with Gleason score ≤ 6 (adjusted OR = 1.24, 95% CI: 1.10–1.41, *P* = 6.53 × 10 ^−4^), Gleason score = 7 (adjusted OR = 1.27, 95% CI: 1.10–1.47, *P* = 1.25 × 10 ^−3^), PSA < 10 ng/mL (adjusted OR = 1.22, 95% CI: 1.09–1.37, *P* = 6.88 × 10 ^−4^), 10 ≤ PSA < 20 ng/mL (adjusted OR = 1.33, 95% CI: 1.10–1.62, *P* = 3.64 × 10 ^−3^), and stage Ⅰ/Ⅱ (adjusted OR = 1.23, 95% CI: 1.10–1.37, *P* = 2.57 × 10 ^−4^). No significant risk was identified in high-risk PCa subgroups, such as Gleason ≥ 8, PSA > 20, and stage Ⅲ/Ⅳ subgroups, compared with the controls.

**Table 2:** Stratification analyses of clinicopathologic variables for the association between rs11973492 and prostate cancer risk in the dominant model

Variables	Genotypes				OR (95% CI) ^a^	*P* ^a^
	TT ( *n*)	%	TC & CC ( *n*)	%		
Controls	1537	49.92	1542	50.08		
Cases	2089	45.18	2535	54.82	1.20 (1.08–1.34)	7.06×10 ^−4^
Gleason score						
≤6	1219	45.16	1480	54.84	1.24 (1.10–1.41)	6.53×10 ^−4^
=7	640	44.51	798	55.49	1.27 (1.10–1.47)	1.25×10 ^−3^
≥8	203	46.88	230	53.12	1.16 (0.94–1.43)	1.71×10 ^−1^
PSA (ng/mL)						
<10	1625	45.18	1972	54.82	1.22 (1.09–1.37)	6.88×10 ^−4^
10–20	254	43.87	325	56.13	1.33 (1.10–1.62)	3.64×10 ^−3^
>20	114	50.89	110	49.11	0.96 (0.72–1.27)	7.58×10 ^−1^
Stages						
Stage Ⅰ/Ⅱ	1813	44.98	2218	55.02	1.23 (1.10–1.37)	2.57×10 ^−4^
Stage Ⅲ/Ⅳ	276	46.62	316	53.38	1.17 (0.95–1.45)	1.32×10 ^−1^
^a^Adjusted for age, smoking status and the top ten principal components in the logistic regression model. Abbreviations: OR, odds ratio; CI, confidence interval; PSA, prostate specific antigen.						

### Annotation of SNPs and expression levels of parental genes

An overview of rs11973492 is presented in ***Supplementary Fig. 1*** (available online). Chromatin immunoprecipitation sequencing data revealed that rs11973492 was located within the binding site of CCCTC-binding factors in prostate epithelial cells ( ***Supplementary Fig. 1A***). The variant of rs11973492 might also affect the secondary structures of the corresponding RNAs ( ***Supplementary Fig. 1B*** and ***1C***). Additionally, several genes ( *EVX1*, *HOTTIP*, *HOXA-AS4*, *HOXA10*, *etc*.) were observed to interact with rs11973492 through three-dimensional chromatin loops in PCa cell lines ( ***Supplementary Fig. 1D***).

Significantly higher expression levels of *circHIBADH* were detected in 25 adjacent normal tissues, compared with paired tumor tissues in the MiOncoCirc database ( *P* = 1.79 × 10 ^−7^, ***Fig. 1C***), while the expression level of *HIBADH*, which is the parental gene of *circHIBADH*, did not differ between adjacent normal and tumor tissues ( *P* = 7.71 × 10 ^−1^, ***Fig. 1E***). In the TCGA and GSE94767 datasets, the differences in expression levels of *HIBADH* between normal and tumor tissues were not significant ( ***Fig. 1F*** and ***1G***), suggesting that *circHIBADH* functioned independently from its parental gene.

### Functional annotation of *circHIBADH*

**Figure 2:**
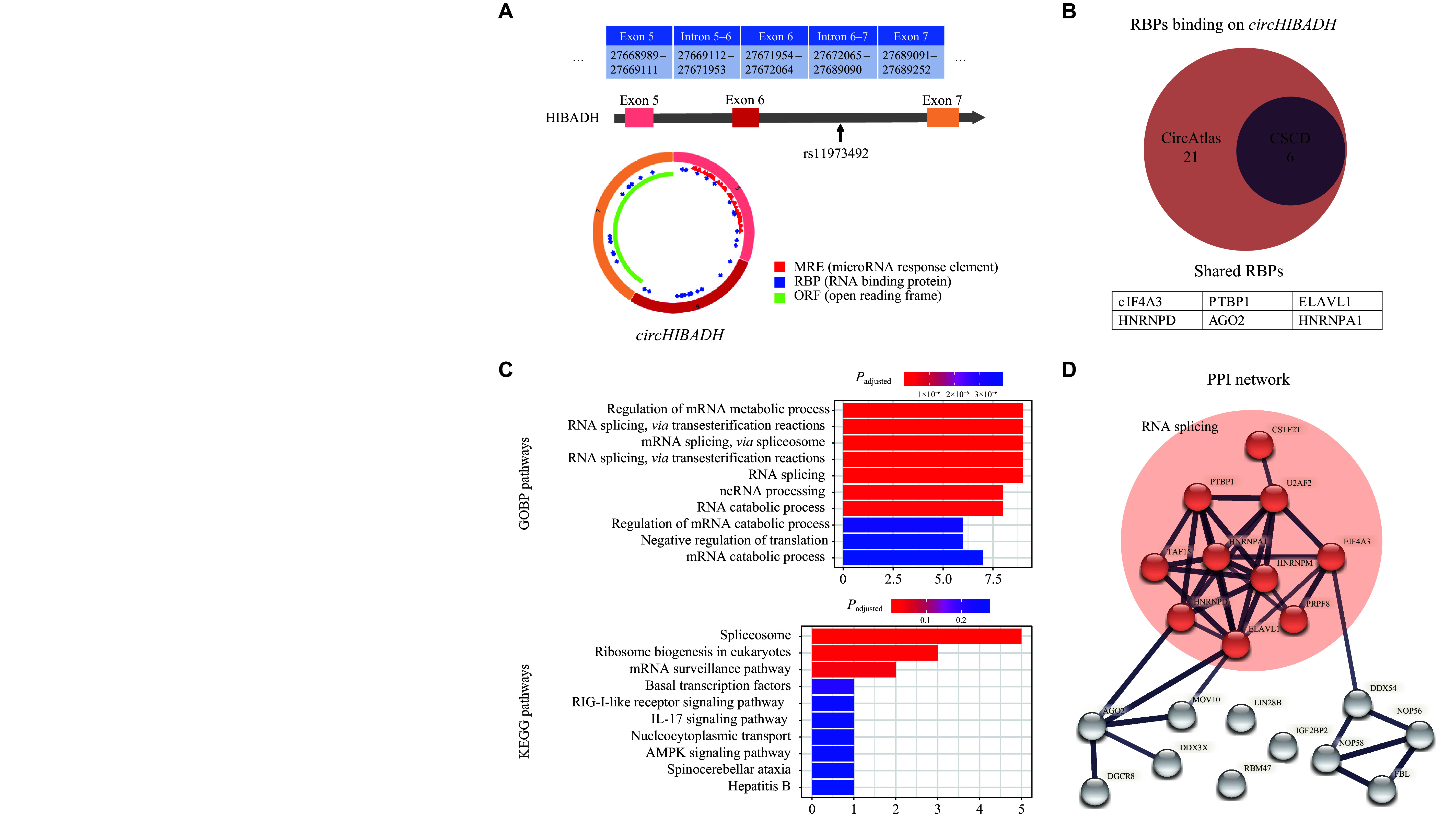
Annotation of *circHIBADH*.

An overview of *circHIBADH* is presented in ***Fig. 2A***. The rs11973492 SNP is located in the intron between exon 6 and exon 7 of *HIBADH*, implying that the variation of rs11973492 may reduce the generation of *circHIBADH* by altering the structure of RNA chains. In total, we predicted 21 potential RBPs of *circHIBADH* in CircAtlas, among which six ( *i*. *e*., eIF4A3, PTBP1, ELAVL1, HNRNPD, AGO2, and HNRNPA1) were also identified in CSCD ( ***Fig. 2B***). Functional annotation implied that these RBPs were predominantly associated with RNA splicing processes ( ***Fig. 2C***). The PPI network also prompted that the interaction of predicted RBPs mainly enriched in the process of RNA splicing, in which proteins HNRNPA1 and HNRNPM appeared to be hubs of the network ( ***Fig. 2D***).

### Identification and validation of hub RBPs

**Figure 3:**
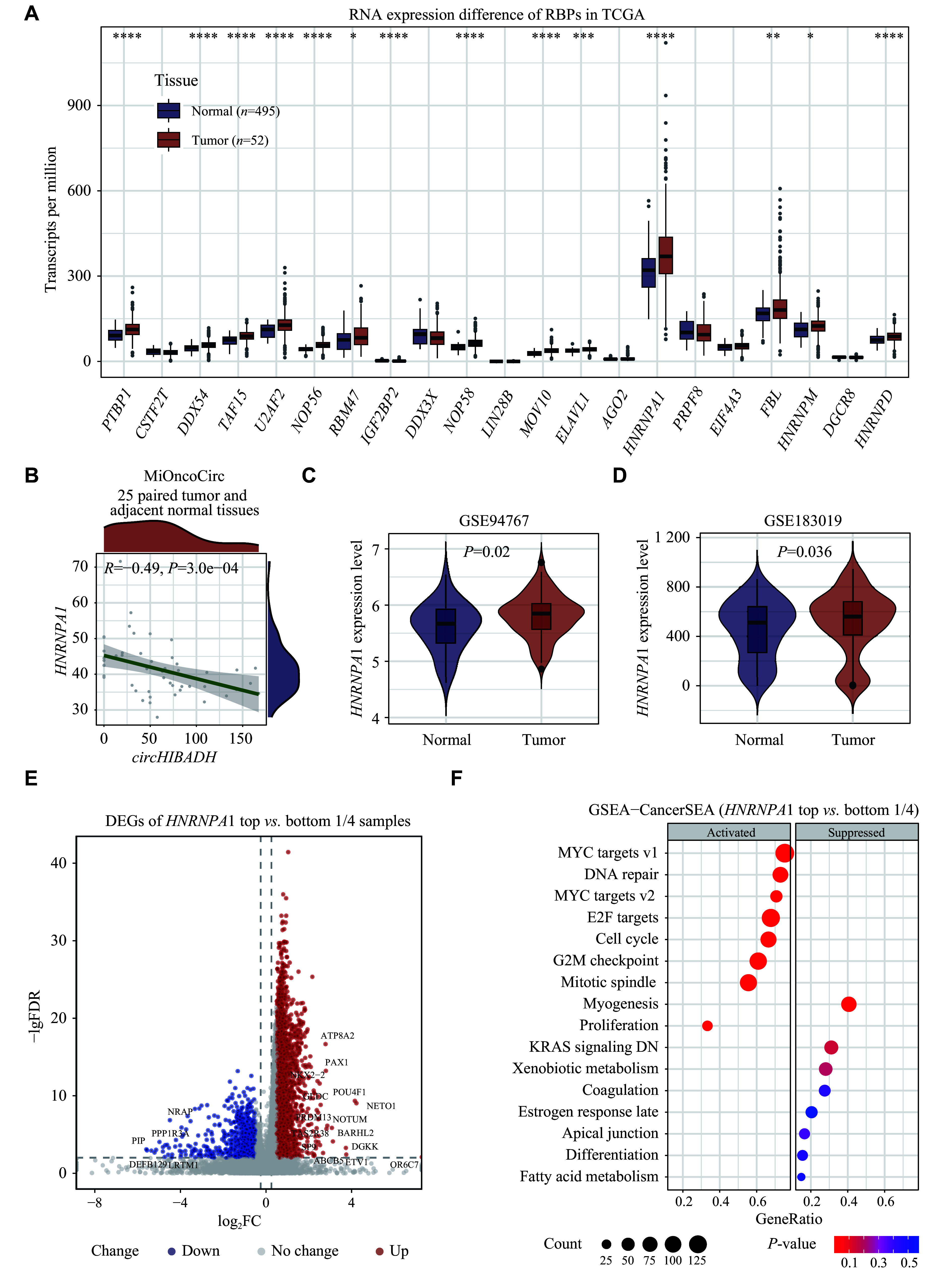
Identification and validation of hub RBP.

We assessed the expression levels of 21 predicted RBPs in the TCGA PRAD dataset, and found that *HNRNPA1* exhibited consistently high expression levels among the tested samples, and was significantly over-expressed in tumor tissues ( *P* = 3.98 × 10 ^−5^, ***Fig. 3A***). We also found a notably negative correlation between *circHIBADH* and *HNRNPA1* expression levels ( *R* = −0.49, *P* = 3.01 × 10 ^−4^, ***Fig. 3B***), implying that *circHIBADH* may function as a protein sponge, thus inhibiting the HNRNPA1-induced RNA splicing process. Integrating with the aforementioned inter-protein interactions, HNRNPA1 was identified as the hub RBP in the *circHIBADH* regulating RNA splicing process. Firstly, we identified that the T allele of rs11973492 was associated with a higher expression level of *HNRNPA1*, although not statistically significant ( *P* = 6.79 × 10 ^−2^, ***Supplementary Fig. 2*** [available online]). In addition, the up-regulation of *HNRNPA1* in tumors was replicated in GSE94767 and GSE183019 datasets ( *P* = 1.99 × 10 ^−2^ and 3.64 × 10 ^−2^, respectively, ***Fig. 3C*** and ***3D***). As shown in ***Fig. 3E***, the DE analysis was performed on samples of the top and the bottom 1/4 *HNRNPA1* expression levels. Moreover, the corresponding GSEA analysis demonstrated that several carcinogenesis-associated hallmarks, *e*. *g*., MYC target signaling, DNA repair, and E2F target signaling, which are known to be correlated with cell proliferation, were found to be activated in the *HNRNPA1*-high tissues ( ***Fig. 3F***). As expectedly, the expression level of *circHIBADH* was significantly negatively correlated with these downstream pathways, including RNA splicing ( ***Supplementary Fig. 3***, available online).

**Figure 4:**
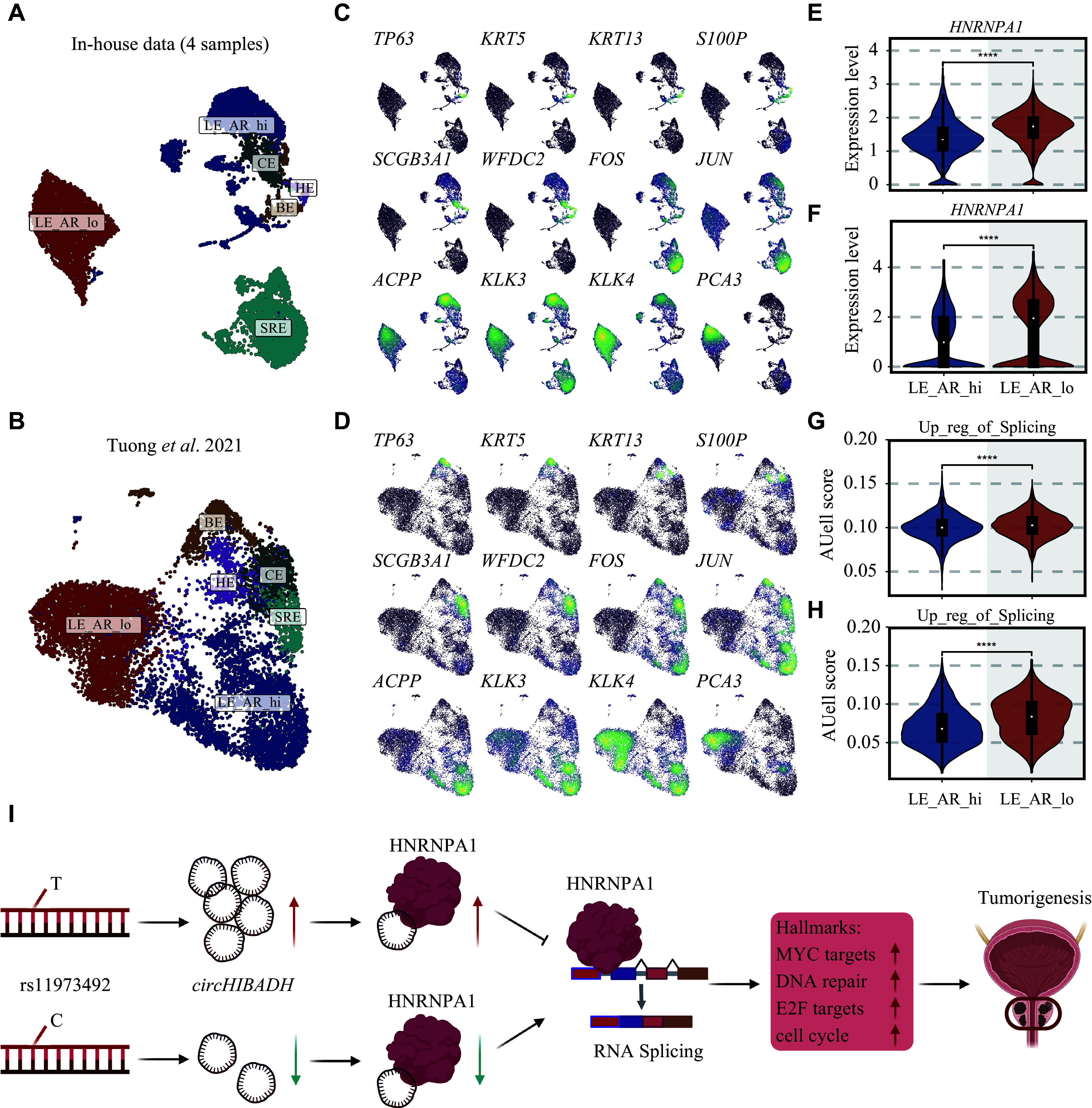
Validation of hub RBP and key biological processes in single-cell transcriptomes.

We then introduced two single-cell transcriptome data for additional validation, of which the cellular landscapes are shown in ***Supplementary Fig. 4*** (available online). Epitheliums were extracted and annotated as basal epithelium ( *TP63* ^+^ *KRT5* ^+^), hillock epithelium ( *KRT13* ^+^ *S100P* ^+^), club epithelium ( *SCGB3A1* ^+^ *WFDC2* ^+^), stress response epithelium ( *FOS* ^hi^ *JUN* ^hi^), luminal epithelium-androgen response-high (LE_AR_hi, *ACPP* ^hi^ *KLK3* ^hi^), and LE_AR-low (LE_AR-lo, *ACPP* ^lo^ *KLK4* ^hi^, ***Fig. 4A***– ***4D***), according to the epithelial subtypes identified by Henry *et* *al* ^[ 22] ^. As shown in ***Fig 4C*** and ***4D***, *PCA3*, an established PCa marker, was highly expressed in the LE_AR_lo subtype, so we assigned LE_AR_lo as tumor cells but LE-AR-hi as normal luminal cells. Consistently, the expression levels of *HNRNPA1* were significantly higher in tumor cells than in normal luminal cells ( ***Fig. 4E*** and ***4F***). Furthermore, the gene ontology biological process "Up-regulation of RNA splicing" was notably activated in malignant cells, compared with normal luminal cells ( ***Fig. 4G*** and ***4H***).

## Discussion

In the current study, we discovered rs11973492 in *circHIBADH* as a significant risk factor for PCa. Moreover, rs11679306 appears to inhibit the generation of *circHIBADH* and suppress the sponging of HNRNPA1 and other RBPs, thus enhancing RNA splicing-induced carcinogenesis ( ***Fig. 4I***).

circRNAs are tissue-specific and function in various biological processes in multiple malignancies ^[ 23] ^, including PCa. In terms of the interaction with RBPs, circRNAs may sponge RBPs to inhibit ^[ 9] ^, scaffold, or recruit RBPs to facilitate ^[ 10– 11]^ and enhance RBP expression to promote ^[ 12]^ protein functions. In PCa, it is reported that has_ *circ_0003258* promotes PCa metastasis through complexing with IGF2BP3 ^[ 24] ^, and that *circTFDP2* facilitated PCa progression by sponging and inhibiting PARP1 ^[ 25] ^. In the current study, we found that *circHIBADH* might suppress the RNA splicing role of HNRNPA1 and other RBPs by acting as a protein sponge.

Additionally, a significantly differential expression of *circHIBADH*, but not the parental genes ( *HIBADH*), was found between normal and tumor tissues, suggesting that circRNA functions independently from mRNAs, which may be modulated by rs11973492. Such a phenomenon has been proposed and discussed in previous studies ^[ 19, 26] ^. Some circRNAs located in the nucleus bind to RNA polymerase, thus regulating the transcription of parental genes ^[ 12] ^. For instance, an excision of the *DOCK1* circRNA contributes to the downregulation of *DOCK1* in epithelial cells ^[ 27] ^. Moreover, we found that rs11973492 was significantly associated with an early-stage PCa but not with a late-stage PCa. This disparity, on the one hand, may be attributed to the relatively smaller sample size of late-stage PCa patients. On the other hand, it suggests that rs11973492 may play a specific role in the oncogenesis of PCa rather than the progression, thus explaining its weaker association with late-stage PCa.

Furthermore, *circHIBADH* is an exonic circRNA, and rs11973492 is located in the intron between circRNA-forming exons. Studies have reported that complementary base pairing of inverted repeats in the intron flanking the exons promotes the generation of circular RNA by approaching the splicing site of circular RNA ^[ 27– 28] ^. Liang *et* *al* ^[ 29]^ showed that micro-introns containing splice sites and short inverted repeats promoted the circulation of intervening exons in cells. Moreover, the secondary structure within pre-mRNAs was shown to enhance circRNA biogenesis by bringing circRNA-forming exons into a close proximity ^[ 30] ^. We found that the variant of rs11973492 changed the secondary structure of the corresponding mRNA chain, which may affect the generation of circRNAs and their biological functions.

As a hub RBP, HNRNPA1 was identified to play a key role in the *circHIBADH*-related PCa risk, through the regulation of RNA splicing process. The dysregulation of RNA splicing is common in PCa, of which a notable example is the androgen receptor splice variant AR-V7 ^[ 31] ^. SF3B2-mediated RNA splicing was also found to drive progression in PCa ^[ 32] ^. HNRNPA1, a member of heterogeneous nuclear ribonucleoproteins, is associated with pre-mRNAs in the nucleus and influences pre-mRNA processing. Likewise, HNRNPA1 promotes oncogenesis by regulating proliferation in several malignancies ^[ 33] ^, which is potentially implicated in enzalutamide resistance and aggressiveness in PCa ^[ 34– 36] ^. Additionally, HNRNPA1 has been reported as a biomarker for early biochemical recurrence of PCa ^[ 37] ^. The genotype-based gene expression analysis is important to explore the function of SNPs; so, through expression quantitative trait loci analysis, we found that the T allele of rs11973492 was associated with a higher expression level of *HNRNPA1*. Biologically, this may represent a negative feedback mechanism that occurs following the silencing of HNRNPA1 by the rs11973492-associated elevation of *circHIBADH*, resulting in the increased transcription of *HNRNPA1*.

Through bioinformatics analysis, we speculated that as a downregulated circRNA in tumors, *circHIBADH* exerted its anticancer effect through its protein sponge function. Once such a mechanism was downregulated, the silenced protein ( *i*. *e*., HNRNPA1) may exert its oncogenic function. The protein-binding function of circRNAs has been extensively explored: they act as sponges, scaffolds, recruiters, or function enhancers of the proteins to exert their effects. Protein sponges, similar to the classic competing endogenous RNA mechanisms, inhibit the function of the corresponding RBPs, as demonstrated in the current study. This mechanism of protein sponges has been reported in various studies involving liver cancer, head and neck squamous cell carcinoma, glioblastoma, among others ^[ 38] ^.

In conclusion, we found that the variant of rs11973492 obstructed the generation of *circHIBADH*, which may act as a protein sponge and inhibitor of HNRNPA1 and other RBPs, consequently enhancing RNA splicing processes and subsequent MYC targeting, DNA repair, and E2F target signaling pathways, to play an oncogenic role in PCa.

## SUPPLEMENTARY DATA

Supplementary data to this article can be found online.
