# Supplementary material for: Genetic variation of circHIBADH enhances prostate cancer risk through regulating HNRNPA1-related RNA splicing
Source: J Biomed Res. 2024 May 29;38(4):358–68. doi: 10.7555/JBR.38.20240030 (PMC11300518; doi:10.7555/JBR.38.20240030)
Supplement: Supplementary file 1 — Supplementary data to this article can be found online. [file jbr-38-4-358-S1.pdf]

Available online at www.jbr-pub.org.cn

Open Access at PubMed Central

Journal of Biomedical Research, 2024 0(000): 1–4

JBR

Supplementary Data

Genetic variation of *circHIBADH* enhances prostate cancer risk through regulating HNRNPA1-related RNA splicing

Yifei Cheng[1,△], Rongjie Shi[2,△], Shuai Ben[1], Silu Chen[1], Shuwei Li[1], Junyi Xin[3], Meilin Wang[4,5,#], Gong Cheng[2,#,✉]

[1]*Department of Environmental Genomics, Jiangsu Key Laboratory of Cancer Biomarkers, Prevention and Treatment, Collaborative Innovation Center for Cancer Personalized Medicine, Nanjing Medical University, Nanjing, Jiangsu 211166, China;*
[2]*Department of Urology, the First Affiliated Hospital of Nanjing Medical University, Nanjing, Jiangsu 210029, China;*
[3]*Department of Bioinformatics, School of Biomedical Engineering and Informatics, Nanjing Medical University, Nanjing, Jiangsu 211166, China;*
[4]*Jiangsu Cancer Hospital, Jiangsu Institute of Cancer Research, the Affiliated Cancer Hospital of Nanjing Medical University, Nanjing, Jiangsu 210009, China;*
[5]*The Affiliated Suzhou Hospital of Nanjing Medical University, Suzhou Municipal Hospital, Gusu School, Nanjing Medical University, Suzhou, Jiangsu 215002, China.*

Supplementary Table 1 (available online) shows the circRNAs detected and differentially expressed in 25 paired prostate tumor and adjacent normal tissues

Supplementary Table 2 **Associations between SNPs and the risk of prostate cancer**

Chr	SNPs	Alleles (Ref/Alt)	P_{hwe}	Call rates	MAF	$MAF_{control}$	MAF_{case}	OR[a]	Lower CI[a]	Upper CI[a]	P[a]	FDRs
7	rs11973492	T/C	0.21	0.99	0.31	0.30	0.32	1.20	1.08	1.34	7.06E−04	4.30E−02
4	rs28553543	T/C	0.91	0.97	0.05	0.05	0.05	0.85	0.72	1.02	7.96E−02	8.69E−01
8	rs6586642	T/C	0.17	0.95	0.15	0.15	0.16	1.11	0.98	1.25	9.05E−02	8.69E−01
1	rs705534	C/A	1.00	1.00	0.15	0.14	0.15	1.11	0.98	1.24	9.78E−02	8.69E−01
4	rs17607518	A/G	0.33	0.99	0.15	0.16	0.15	0.91	0.81	1.03	1.25E−01	8.69E−01
6	rs1207037	T/C	0.11	0.98	0.11	0.10	0.11	1.11	0.97	1.26	1.37E−01	8.69E−01
4	rs73212864	G/A	0.75	1.00	0.23	0.22	0.23	1.08	0.97	1.21	1.41E−01	8.69E−01
4	rs3733415	G/A	0.05	1.00	0.14	0.13	0.14	1.09	0.97	1.23	1.54E−01	8.69E−01
4	rs13149290	C/T	0.95	1.00	0.23	0.23	0.22	0.93	0.83	1.03	1.79E−01	8.69E−01
1	rs834487	G/A	0.24	1.00	0.07	0.07	0.07	1.11	0.95	1.29	1.86E−01	8.69E−01
4	rs56079809	C/A	0.07	0.97	0.20	0.20	0.20	1.08	0.96	1.20	1.99E−01	8.69E−01
2	rs11680405	T/C	0.46	0.97	0.32	0.32	0.33	1.07	0.96	1.19	2.11E−01	8.69E−01
7	rs2110524	T/G	0.01	1.00	0.07	0.07	0.07	1.10	0.94	1.28	2.41E−01	8.69E−01
8	rs2979788	A/G	0.70	1.00	0.16	0.16	0.16	1.07	0.95	1.20	2.41E−01	8.69E−01
7	rs62454908	T/C	0.51	0.99	0.24	0.25	0.23	0.94	0.84	1.05	2.45E−01	8.69E−01
8	rs209568	T/C	0.20	0.99	0.22	0.21	0.22	1.07	0.96	1.19	2.53E−01	8.69E−01
6	rs12195875	A/G	0.13	1.00	0.19	0.19	0.19	1.07	0.95	1.19	2.67E−01	8.69E−01
7	rs59505903	C/T	0.85	0.99	0.14	0.13	0.14	1.06	0.94	1.20	3.54E−01	8.69E−01

△These authors contributed equally to this work.
#These authors jointly supervised this work.

✉Corresponding author: Gong Cheng, Department of Urology, the First Affiliated Hospital of Nanjing Medical University, 300 Guangzhou Road, Nanjing, Jiangsu 210029, China. E-mail: gcheng@njmu.edu.cn.

Received: 02 February 2024; Revised: 24 April 2024; Accepted: 30 April 2024; Published online: 29 May 2024

CLC number: R737.25, Document code: A

The authors reported no conflict of interests.

This is an open access article under the Creative Commons Attribution (CC BY 4.0) license, which permits others to distribute, remix, adapt and build upon this work, for commercial use, provided the original work is properly cited.

© 2024 by Journal of Biomedical Research.

https://doi.org/10.7555/JBR.38.20240030

Supplementary Table 2 **Associations between SNPs and the risk of prostate cancer** (continued)

Chr	SNPs	Alleles (Ref/Alt)	P_{hwe}	Call rates	MAF	$MAF_{control}$	MAF_{case}	OR[a]	Lower CI[a]	Upper CI[a]	P[a]	FDRs
4	rs933823	G/A	0.55	0.99	0.32	0.31	0.32	1.05	0.94	1.17	3.93E−01	8.69E−01
4	rs12646870	C/T	0.18	1.00	0.49	0.49	0.49	1.05	0.93	1.19	3.95E−01	8.69E−01
7	rs12674175	G/A	0.22	0.99	0.11	0.12	0.11	0.95	0.83	1.08	3.96E−01	8.69E−01
2	rs12479331	G/A	0.41	0.99	0.24	0.25	0.24	0.96	0.86	1.06	4.01E−01	8.69E−01
6	rs3800003	C/T	0.68	1.00	0.21	0.20	0.21	1.05	0.94	1.17	4.06E−01	8.69E−01
6	rs214507	T/C	0.81	0.99	0.21	0.21	0.21	0.96	0.86	1.07	4.24E−01	8.69E−01
2	rs13391923	C/T	0.14	1.00	0.08	0.08	0.08	1.06	0.91	1.23	4.37E−01	8.69E−01
4	rs34893238	C/G	0.08	0.99	0.30	0.29	0.31	1.04	0.94	1.16	4.67E−01	8.69E−01
5	rs3733876	G/A	0.22	1.00	0.18	0.18	0.18	1.04	0.93	1.16	5.25E−01	8.69E−01
2	rs7591409	T/C	0.07	1.00	0.12	0.12	0.12	0.96	0.85	1.09	5.30E−01	8.69E−01
7	rs2230585	G/A	0.58	1.00	0.38	0.38	0.38	1.04	0.93	1.15	5.34E−01	8.69E−01
4	rs16994217	C/T	0.33	0.99	0.13	0.13	0.13	0.96	0.85	1.09	5.36E−01	8.69E−01
4	rs55941493	G/A	0.49	0.99	0.47	0.47	0.46	0.96	0.86	1.08	5.44E−01	8.69E−01
4	rs2114014	A/G	0.18	0.98	0.35	0.35	0.35	0.97	0.87	1.08	5.50E−01	8.69E−01
1	rs834477	G/A	0.19	0.99	0.22	0.21	0.22	1.03	0.93	1.15	5.51E−01	8.69E−01
4	rs17020674	G/C	0.43	1.00	0.29	0.29	0.29	1.03	0.93	1.15	5.68E−01	8.69E−01
1	rs766234	T/G	0.86	0.98	0.35	0.35	0.34	0.97	0.87	1.08	5.70E−01	8.69E−01
2	rs11889931	C/T	0.53	0.98	0.38	0.38	0.38	1.03	0.93	1.15	5.70E−01	8.69E−01
6	rs214508	T/C	0.56	1.00	0.35	0.34	0.35	1.03	0.93	1.15	5.73E−01	8.69E−01
6	rs214509	G/C	0.85	1.00	0.43	0.43	0.43	0.97	0.87	1.08	5.80E−01	8.69E−01
1	rs17540601	C/T	0.05	0.98	0.12	0.12	0.12	1.03	0.91	1.17	6.18E−01	8.69E−01
1	rs12567062	G/A	0.79	0.96	0.41	0.41	0.40	0.97	0.87	1.09	6.31E−01	8.69E−01
7	rs73729985	C/T	0.49	1.00	0.06	0.06	0.06	0.96	0.82	1.13	6.40E−01	8.69E−01
7	rs13231645	C/G	0.91	1.00	0.17	0.18	0.17	0.98	0.87	1.09	6.67E−01	8.69E−01
8	rs3739407	G/A	0.10	1.00	0.19	0.19	0.18	0.98	0.87	1.09	6.68E−01	8.69E−01
4	rs2241487	A/C	0.64	0.99	0.07	0.06	0.07	0.97	0.82	1.14	6.85E−01	8.69E−01
2	rs4012753	A/C	0.78	1.00	0.07	0.06	0.07	1.03	0.88	1.21	6.91E−01	8.69E−01
4	rs13129325	C/G	1.00	0.99	0.07	0.07	0.07	0.97	0.83	1.14	7.20E−01	8.69E−01
2	rs10208940	T/C	0.77	1.00	0.14	0.14	0.14	1.02	0.91	1.15	7.22E−01	8.69E−01
4	rs34104130	G/T	0.33	0.99	0.26	0.27	0.26	0.98	0.88	1.09	7.28E−01	8.69E−01
2	rs11678171	C/T	0.19	1.00	0.15	0.15	0.15	1.02	0.91	1.15	7.50E−01	8.69E−01
7	rs6467405	G/T	0.59	0.97	0.34	0.33	0.35	1.02	0.91	1.13	7.51E−01	8.69E−01
4	rs717614	C/G	0.71	0.97	0.47	0.48	0.46	0.98	0.87	1.11	7.55E−01	8.69E−01
1	rs61542269	C/A	0.31	0.98	0.24	0.25	0.24	0.98	0.88	1.10	7.57E−01	8.69E−01
8	rs56026721	C/A	0.51	0.95	0.07	0.07	0.07	0.98	0.83	1.14	7.57E−01	8.69E−01
8	rs2013586	A/G	0.66	1.00	0.41	0.42	0.41	0.98	0.88	1.10	7.69E−01	8.69E−01
4	rs10434448	C/G	0.28	1.00	0.22	0.22	0.22	0.99	0.89	1.10	8.19E−01	9.08E−01
4	rs1013842	A/G	0.81	1.00	0.11	0.11	0.11	1.01	0.89	1.15	8.69E−01	9.47E−01
6	rs3800004	A/G	0.61	0.95	0.32	0.32	0.32	1.01	0.90	1.12	9.03E−01	9.54E−01
2	rs1025869	C/A	0.88	1.00	0.08	0.08	0.08	1.01	0.87	1.17	9.07E−01	9.54E−01
4	rs9790518	T/C	0.13	1.00	0.41	0.40	0.41	0.99	0.89	1.11	9.26E−01	9.55E−01
8	rs3739408	T/C	0.26	1.00	0.14	0.14	0.14	1.00	0.88	1.12	9.47E−01	9.55E−01
4	rs12499240	C/T	0.48	0.98	0.27	0.27	0.27	1.00	0.90	1.12	9.55E−01	9.55E−01

[a]Adjusted for age, smoking status, and the top ten principal components in the logistic regression model. Abbreviations: HWE, Hardy-Weinberg equilibrium; MAF,

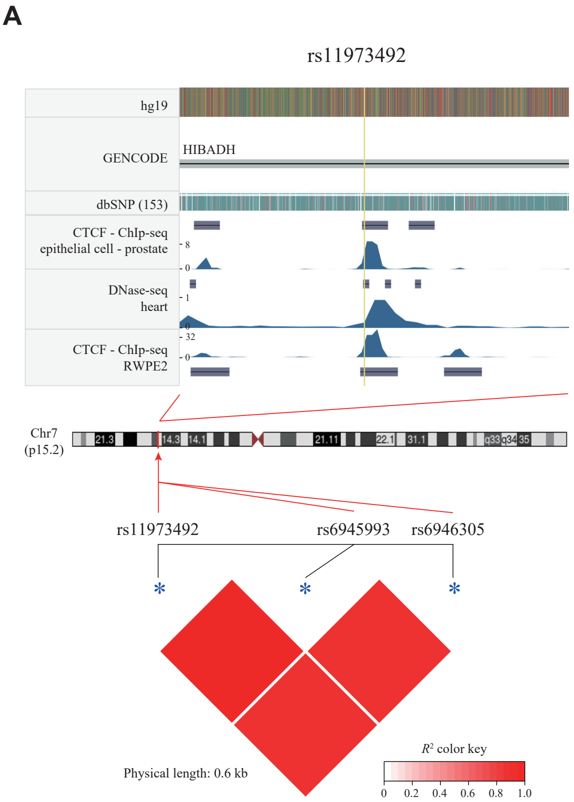

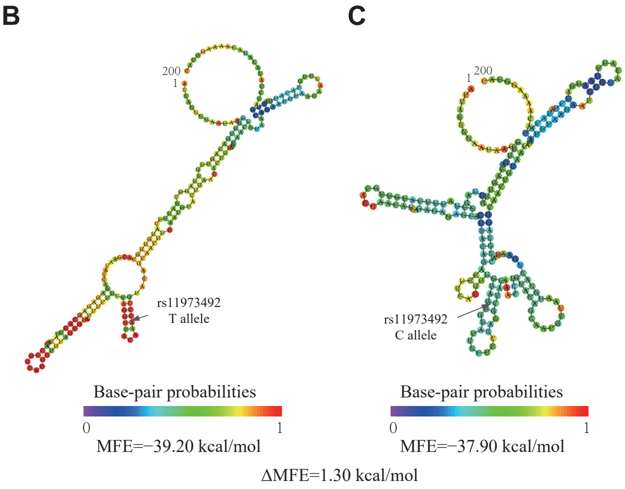

D

Genes that interact rs11973492 through 3D chromatin loops in PCa cell lines

Interact genes	Cell type	Loop start	Loop end	Distance (bp)
EVX1				
HOTTIP				
HOXA-AS4				
HOXA10				
HOXA10-HOXA9	LNCaP /PC3	Chr7: 27 200 000–27 300 000	Chr7: 27 600 000–27 700 000	400 000
HOXA11				
HOXA11-AS				
HOXA13				
HOXA9				
MIR196B				

Supplementary Fig. 1 **Annotation of rs11973492.** A: The genomic loci and clump of rs11973492. B and C: Changes in the secondary structure and minimum free energy (MFE) with rs11973492 T > C allele. D: Genes that interact rs11973492 through three-dimensional chromatin loops in PCa cell lines.

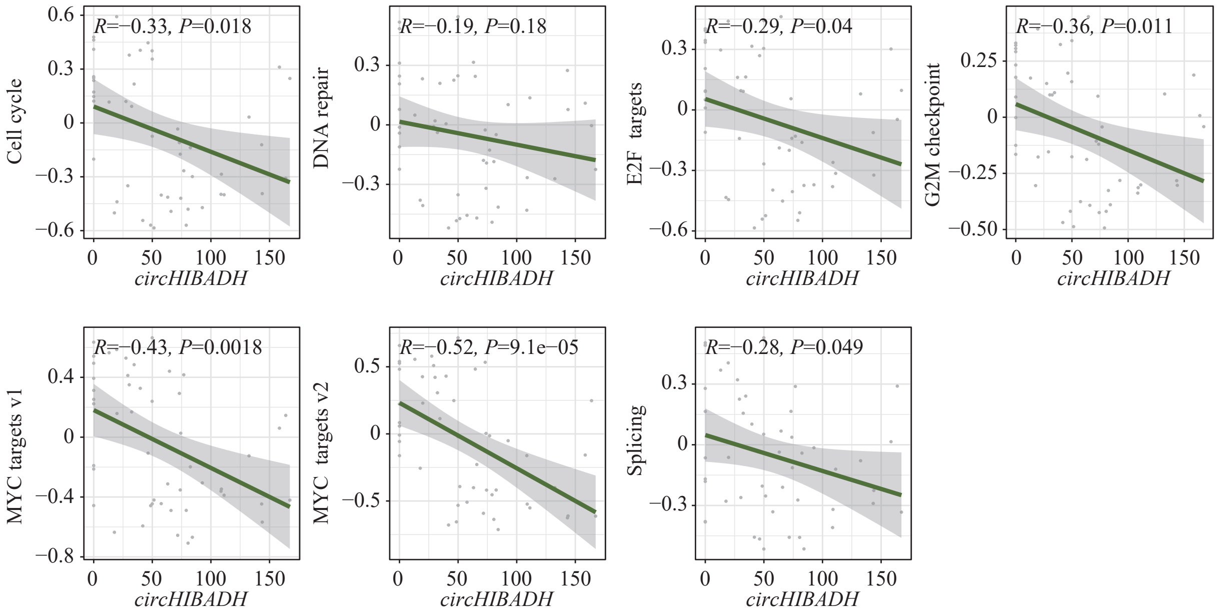

Supplementary Fig. 2 **The correlation between expression levels of *circHIBADH* and the downstream signaling pathways.**

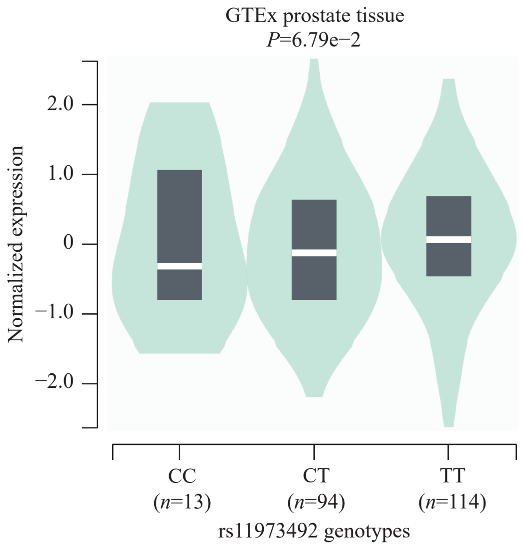

Supplementary Fig. 3 **The expression quantitative trait loci analysis of rs11973492 to HNRNPA1 in prostate tissues based on the GTEx database.** Abbreviation: GTEx, Genotype-Tissue Expression.

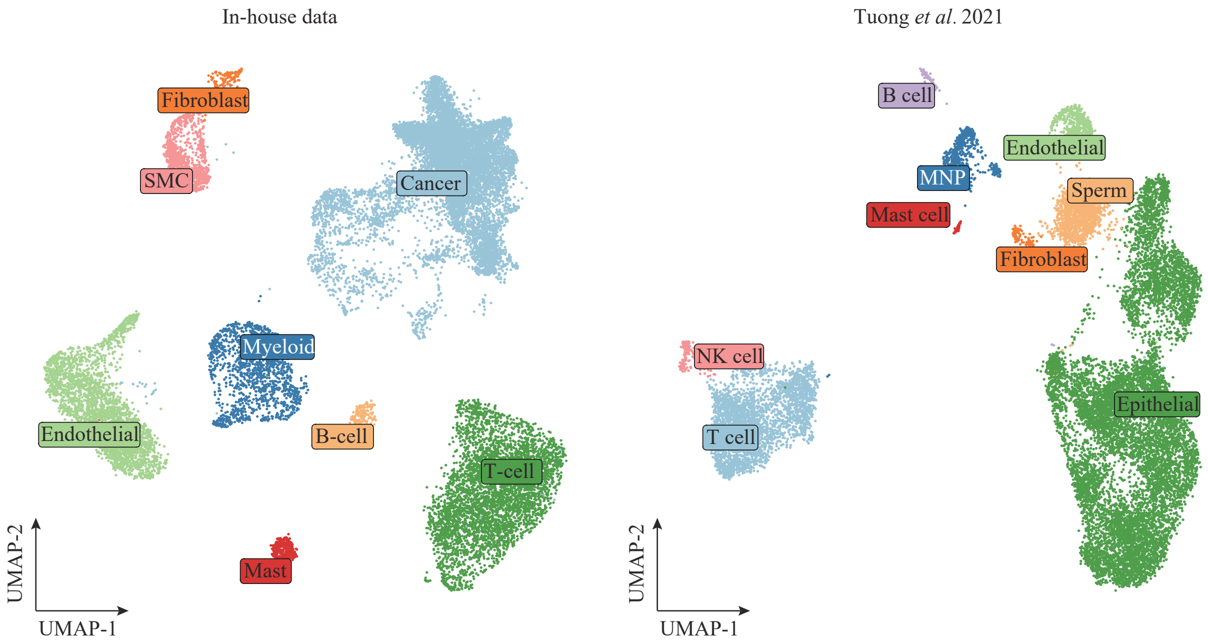

Supplementary Fig. 4 **UMAP plot showing the landscape of PCa tissue cells from in-house data (Left) and Tuong *et al* (Right).** Abbreviations: UMAP, Uniform Manifold Approximation and Projection; SMC, smooth muscle cell; NK, natural killer.
